# Oral Administration of Red Ginseng Extract Promotes Neurorestoration after Compressive Spinal Cord Injury in Rats

**DOI:** 10.1155/2017/1265464

**Published:** 2017-07-30

**Authors:** Pengxiang Zhu, Keiichi Samukawa, Hiroko Fujita, Hidemasa Kato, Masahiro Sakanaka

**Affiliations:** Department of Functional Histology, Ehime University Graduate School of Medicine, Ehime, Japan

## Abstract

Red ginseng and its active ingredients have been shown to decrease neuron death after brain ischemia in experimental animals. However, little is known about the effects of orally administered ginseng extract on spinal cord injury. We orally gave red ginseng extract (RGE) to rats with compressed spinal cord injury (SCI). Open-field locomotor scores were measured as indices of motor function. Histopathological changes and cytokine expressions in situ after SCI were evaluated. Compared to vehicle treatment, RGE treatment (350 mg/kg/day) significantly improved locomotor score up to levels close to those pre-SCI, prevented neuron loss, and facilitated the restoration of white matter in the spinal cord at 14 days after SCI. Treatment with RGE caused less aggregation of Iba-1-positive microglia in grey and white matter at 7 days after SCI, upregulated the expression levels of VEGF and Bcl-xL, and reduced IL-1*β* and TNF*α* expressions in the spinal cord at 7 and 14 days after SCI. We concluded that oral administration of RGE facilitates almost complete functional recovery from motor and behavioral abnormalities in rats with SCI and prevents neuron death in situ, possibly through inhibition of inflammation and upregulation of neuroprotective factors in the injured spinal cord.

## 1. Introduction

The main cause of spinal cord injury (SCI) is trauma associated with motor-vehicle accidents, sport and recreational activities, work-related accidents, and falls at home. Traumatic injuries to the spinal cord result in degeneration of neurons and extensive axonal loss [1]. Several mechanisms appear to be involved in spinal cord degeneration after SCI. To be more specific, mechanical injury to the spinal cord causes initial damage to neurons and axons, and secondary damage can be induced by the ensuing inflammatory responses [2, 3], loss of blood supply, and excess release of excitatory neurotransmitters [4]. Desperate efforts were made to develop the means of effective therapy for SCI. Presently, surgical decompression of the spinal cord, stabilization of the vertebrae, and intravenous administration of methylprednisolone are performed to prevent further injury after SCI, resulting in limited recovery from functional loss [5, 6]. Since the primary damage caused by trauma is irreversible, strategies for finding effective treatment should focus on prevention and reduction of neuron loss from secondary damage after SCI, as well as promoting regeneration of damaged axons in the long term.

Red ginseng root (*Panax ginseng* C. A. Meyer) has been used clinically or empirically in Asian countries for the treatment of various diseases including cerebrovascular disease, hypertension, atherosclerosis, liver dysfunction, and postmenopausal disorders. Our previous studies demonstrated protective effects of ginseng and its ingredients on the central nervous system under ischemic insult [7, 8]. We further showed that ginsenoside Rb1 (gRb1), a main saponin ingredient of red ginseng root, protects ischemic brain through upregulation of the expression of Bcl-x_L_ in vitro and in vivo [9]. Despite considerable information concerning the effects of ginseng and its extracts on brain ischemia [7–9], there are only a few reports about the in vivo actions of red ginseng extract in spinal cord injury. Kim et al. [10] reported that intraperitoneally injected ginseng extracts improved recovery after contusive spinal cord injury on rats. Compared to intraperitoneal injection, oral administration of ginseng extract is presumably free from side effect and likely to be accepted by patients since it is the traditional way to take red ginseng. In the present study, we showed that oral administration of red ginseng extract (RGE) promotes recovery from motor and behavioral abnormalities in rats with SCI. Subsequent histopathological observations using MAP2 and Iba-1 immunostaining, and analyses of IL-1*β*, TNF*α*, VEGF, and Bcl-x_L_ expressions demonstrated that RGE promotes neuronal restoration in the injured spinal cord by inhibiting the inflammatory processes and by upregulating the expression of neuroprotective factors (VEGF and Bcl-x_L_) after SCI.

## 2. Materials and Methods

All experiments were approved by the Ethics Committee of Ehime University School of Medicine and were conducted according to the Guidelines for Animal Experimentation at Ehime University School of Medicine.

Animals were housed in an animal room with a temperature range of 21 to 23°C and a 12-hour light/dark cycle (light on: 7 a.m. to 7 p.m.), with access to food and water ad libitum until the end of the experiment.

### 2.1. Oral Administration of Red Ginseng Extracts

**Figure 1:**
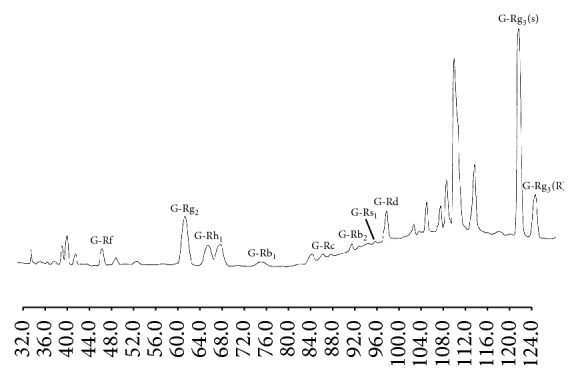
HPLC separation of ginsenosides in red ginseng extract. Column: Superspher RP-18(e) (4.0 mm i.d. × 250 mm, Merck); mobile phase: (A) CH_3_CN-H_2_O-0.1% H_3_PO_4_ (21 : 72 : 8, v/v), (B) CH_3_CN; flow rate: total flow 0.8 ml/min (flow program: (A) 0 → 19 min: 100%; 19 → 20 min: 100 → 90%; 20 → 73 min: 90%; 73 → 103 min: 90 → 70%; 103 → 120 min: 70%); Temp.: Temp. program (0 → 30 min: 35°C; 30 → 60 min: 55°C; 60 → 120 min: 35°C); detection: UV 202 nm.

One hundred and eight adult male Wistar rats, aged 12–14 weeks, weighing 250–300 g, were randomly divided into three groups, a control and two experimental groups. Red ginseng extract (RGE) weighing 100 g as a third-class OTC drug was imported from Korea Ginseng Corporation (http://seikansho.com/drug.html#c). According to the attached document on the drug, 380 g of water-soluble extract can be obtained from 1 kg of ginseng root (*Panax ginseng* C. A. Meyer) cultivated for 6 years. As a result of high performance liquid chromatography (HPLC) analysis [11], we confirmed that the extract contains main ginsenosides such as ginsenosides Rf, Rg_2_, Rh_1_, Rb_1_, Rc, Rb_2_, Rs_1_, Rd, and Rg_3_ (Figure 1).

After being lyophilized, RGE was dissolved in distilled water (DDW) at a concentration of 90 or 9 mg/ml prior to use. Two experimental doses of RGE, 350 mg/day/kg for high dose and 35 mg/kg/day for low dose, or the same volume of DDW for the control group was orally administered to rats once a day for one week before SCI and for two weeks after SCI. Each animal was given 1 ml of RGE solution or the same volume of DDW every day by using an oral sonde to ensure the right doses. The doses of RGE were determined on the basis of studies by Van Kampen et al. [12] and Kim et al. [13], which described the effects of ginseng extracts on experimental animals exposed to ultraviolet and MPTP, respectively.

### 2.2. Spinal Cord Injury (SCI) in Wistar Rat

Spinal cord injury was induced as described elsewhere [14]. In brief, a longitudinal incision was made from the mid to low thoracic vertebrae on the back of the rat after the animal was anesthetized with 1.5% halothane in a 4 : 3 mixture of nitrous oxide and oxygen. The dorsal surface of the lower thoracic cord (Th12) was exposed by laminectomy, and the dura was left intact. A 20 g weight was put on the exposed Th12 extradurally for 20 minutes to induce compressed injury. After the weight was removed, the muscles and skin were sutured with 4-0 silk. The body temperature was kept at 37.0 ± 0.2°C during surgery. This method induces temporary paralysis of the lower extremities in a reproducible manner [15]. High and low doses of RGE were administered orally to rats once a day for 1 week before SCI and for 2 weeks after SCI as described above. For control animals, same volume of DDW (vehicle) was administered.

### 2.3. Behavioral Evaluation

The open-field test was conducted as described previously [14]. In brief, rats were allowed to move freely in the open field (30 × 30 × 30 cm) for 20 min in the light condition. Infrared beams were set on 2 cm above the floor at 10 cm intervals on each X and Y bank of the open field, making a flip-flop circuit between the beams. The total number of circuit breaks was counted as locomotor activity. On the X bank, 11 infrared beams were attached 12 cm above the floor at 2.5 cm intervals, and the total number of beam crossings was counted as rearing activity. The open-field locomotor scores (locomotor activity, rearing activity, and Basso, Beattie, and Bresnahan (BBB) score [16]) were measured before SCI loading, just after the SCI loading, and from 1 day to 14 days after SCI, as indices of motor function. BBB score of sham-operated rats was 21.

### 2.4. Immunohistochemical Staining

After the evaluation of behavioral performance, the animals were deeply anesthetized with chloral hydrate and fixed with 4% paraformaldehyde in 0.1 M phosphate buffer (pH 7.4) at 7 or 14 days after SCI. The damaged spinal cord (1 cm length) was dissected out, embedded in paraffin, and cut to make 5 *μ*m thick serial sections. After being treated with 10% nonimmunized goat serum, each deparaffinized tissue section was incubated with first antibodies (anti-MAP2, SMI 52, Sternberger Monoclonals Inc., Lutherville, MD, USA; anti-Iba-1, WAKO, Osaka, Japan) overnight at 4°C. After incubation with biotinylated second antibodies and ABC mix (VECTASTAIN Elite ABC Kit, Vector Laboratories Inc., CA, USA), staining was visualized with 0.05% 3,3′-diaminobenzidine tetrahydrochloride and 0.01% hydrogen peroxide.

The MAP2-positive cells in five 0.25 mm × 0.25 mm fields in the ventral horns of the spinal cord were counted. For each animal, numbers of MAP2-positive cells were determined in two coronal sections. The mean number of positive cells per unit area (per mm^2^) was calculated in all animals and expressed as mean ± SD.

For quantitative analysis of Iba-1 immunoreactivity in the injured spinal cord, 5 sections of the injured spinal cord in each animal were randomly selected. The intensity of all Iba-1 immunoreactive structures was evaluated on the basis of a relative optical density (ROD) [17]. The ROD of whole field was measured, and the ROD level of background staining was subtracted from that of immunoreactive structures. The result was showed as relative % of control level.

### 2.5. Enzyme-Linked Immunosorbent Assay (ELISA)

Animals were sacrificed at 1, 3, 7, and 14 days after SCI, and, 1 day before SCI, the damaged and control spinal cords (1 cm length) were dissected out and homogenized on ice with radioimmunoprecipitation assay (RIPA) buffer (0.1% SDS, 1.0% Triton-X100, 0.5% sodium deoxycholate, 150 mM sodium chloride, 50 mM Tris, pH 8.0). After being sonicated and centrifuged, the protein content in the supernatant of the sample was determined using a BCA protein assay kit (Pierce, Rockland, IL, USA). The final protein concentration of 1 mg/ml was made by mixing the supernatant with PBS buffer. TNF*α* and IL-1*β* protein levels were determined using a rat TNF*α* ELISA kit (RTA00, R&D Systems, Minneapolis, MN, USA) and a rat IL-1*β* ELISA kit (SRLB00, R&D Systems, Minneapolis, MN, USA). Measurements were performed according to the manufacturer's instructions.

### 2.6. Immunoblotting Study

The preparation of the sample for Western blotting was described previously [14]. In brief, animals were sacrificed at 7 and 14 days after SCI and 1 day before SCI. The damaged and control spinal cords (1 cm length) were dissected out and homogenized on ice with RIPA buffer. After being sonicated and centrifuged, the protein content in the supernatant of the sample was determined using a BCA protein assay kit (Pierce, Rockland, IL, USA). The final protein concentration of 1 mg/ml was made by mixing the supernatant with sample buffer (62.5 mM Tris-HCl, pH 6.8, 2% sodium dodecylsulfate, 10% glycerol, and 0.001% bromophenol blue). Equal amounts of protein (15 *μ*g) from the homogenates were electrophoresed and processed for immunoblot analysis using antibodies against VEGF (Santa Cruz Biotech, Santa Cruz, CA, USA) and Bcl-x_L_ (Transduction Laboratories Inc., Lexington, KY, USA). An NIH Image program (National Institutes of Health, Bethesda, MD, USA) was used for semiquantitative analysis of the immunoreactive bands.

### 2.7. Statistics

All values are presented as mean value ± SD. Statistical significance was tested by one-way ANOVA followed by Bonferroni's multiple comparison test. A *p* value less than 0.05 was considered statistically significant.

## 3. Results

### 3.1. Oral Administration of RGE Promotes Restoration from Motor and Behavioral Abnormalities in Rats at 2 Weeks after SCI

**Figure 2:**
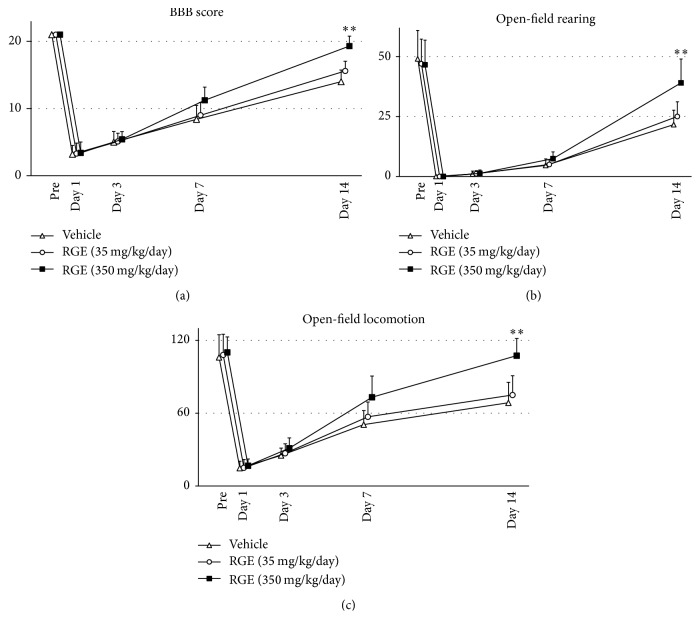
Oral administration of RGE improved BBB score, rearing activity, and locomotion activity at 14 days after spinal cord injury (SCI) in rats. There was the significant improvement of three functional parameters in (350 mg/kg/day) RGE-treated rats (*n* = 5 in each group) at 14 days after SCI. All values are presented as mean value ± SD. ^*∗∗*^(*p* < 0.01) indicates significantly higher values than vehicle-treated control.

We first investigated the effects of oral administration of RGE on BBB score (Figure 2(a)), rearing activity (Figure 2(b)), and locomotion activity (Figure 2(c)) in rats. All animals exhibited motor and behavioral abnormalities after SCI and survived until the end of the experiments. Compared with the vehicle-treated group, the high-dose (350 mg/kg/day) RGE-treated group showed significant improvement of motor and behavioral abnormalities at 14 days after SCI (*n* = 5 in each group). BBB score, open-field rearing, and open-field locomotion in the high-dose RGE-treated group reached values close to those in pre-SCI animals by 14 days after SCI (Figure 2). This suggests that oral administration of RGE facilitates almost complete functional recovery from the damage induced by SCI. The low dose of RGE (35 mg/kg/day, *n* = 5) did not exhibit any significant effects on SCI-induced motor and behavioral abnormalities. Based on the above results of functional experiments, we concentrated on comparing the high-dose RGE-treated group with the vehicle-treated group in all experiments conducted hereafter.

### 3.2. Oral Administration of RGE Ameliorates Morphological Damage to Spinal Cord in Rats at 2 Weeks after SCI

**Figure 3:**
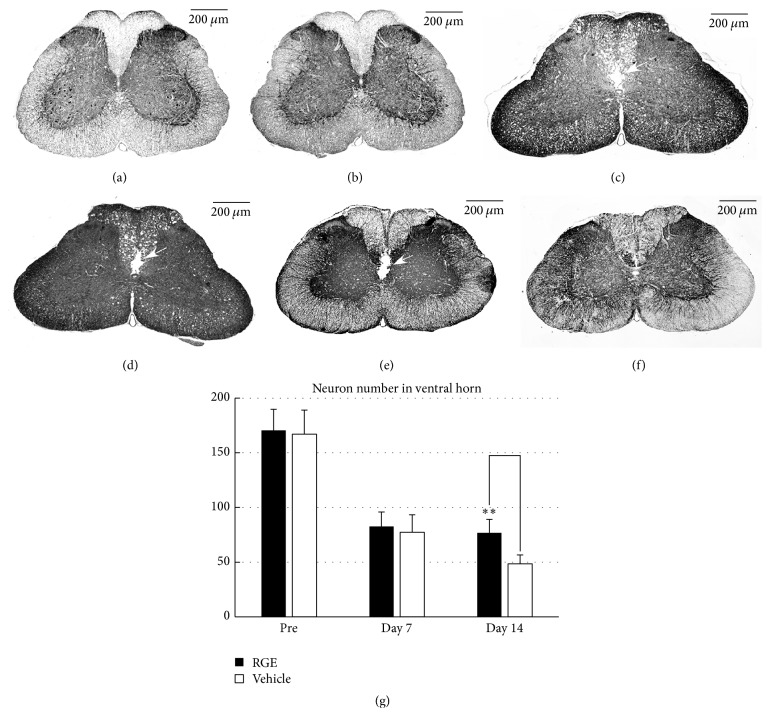
Oral administration of RGE ameliorated morphological damage to spinal cord at 14 days after SCI in rats. (a–f) Representative photomicrographs of MAP2 immunostaining in sections from spinal cords of pre-SCI rats and damaged spinal cords of rats at 7 and 14 days after SCI ((a), (c), and (e): vehicle (DDW); (b), (d), and (f): RGE (350 mg/kg/day); (a) and (b): pre-SCI; (c) and (d): 7 days after SCI; (e) and (f): 14 days after SCI). Scale bar = 200 *μ*m. Cracks are observed in the white matter in and around the central canal ((c), (d), and (e), arrows). (g) Quantification of MAP2-positive cells in the ventral horn of injured spinal cord. There was a significant increase in neural density at day 14 in RGE-treated rats (*n* = 5 in each group). All values are presented as mean ± SD. ^*∗∗*^(*p* < 0.01) indicates significantly higher values than vehicle-treated control.

We next performed immunostaining to evaluate morphological damage after SCI (Figure 3). In pre-SCI rats, the (350 mg/kg/day) RGE-treated and vehicle-treated groups showed similar patterns of MAP2 immunoreactivity in the spinal cord (Figures 3(a) and 3(b)). In the spinal cord of rats at 7 days after SCI, both the vehicle-treated (Figure 3(c)) and RGE-treated (Figure 3(d)) groups exhibited atrophy of grey matter presumably due to the dorsal to ventral compression, a wide and irregular shaped crack, and many vacuoles in the dorsal funiculus (Figures 3(c) and 3(d), open arrow). In the spinal cord of vehicle-treated rats at 14 days after SCI, each crack noted one week previously was still present and appeared to be enlarged as a result of destruction of the central canal and adjacent grey and white matter (Figure 3(e), open arrow). Oral administration of RGE at 350 mg/kg/day prevented such degenerative processes to possibly keep the central canal intact and to markedly reduce the crack size (Figure 3(f)).

To quantify the neuronal damage caused by SCI, MAP2-positive cells per mm^2^ in the ventral horn of each group were counted (Figure 3(g)), and the results demonstrated that MAP2-positive cells were decreased equally in number at 7 days after SCI in the RGE-treated and vehicle-treated groups when compared with those in pre-SCI animals. In the RGE-treated group, the number of MAP2-positive cells was maintained at a constant level even at 14 days after SCI, while, in the vehicle-treated group, MAP2-positive cells at 14 days after SCI were less numerous than those at 7 days after SCI. Consequently, there was a significant difference in MAP2 cell number between the RGE-treated and vehicle-treated groups at 14 days after SCI (Figure 3(g)). The findings obtained from MAP2 immunostaining may provide, in part, a morphological basis for the functional recovery by RGE of rats with SCI.

### 3.3. Oral Administration of RGE Inhibits Inflammatory Responses in Spinal Cord of Rats after SCI

**Figure 4:**
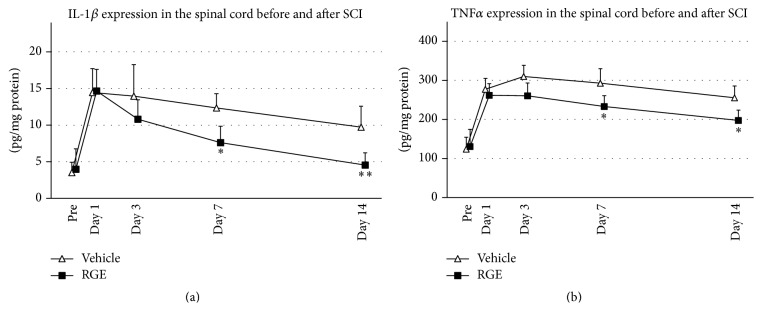
Oral administration of RGE reduced IL-1*β* and TNF*α* expressions in the spinal cord at 7 and 14 days after SCI. (a) Expression of IL-1*β* protein in spinal cord of rats before and after SCI. Compared to vehicle-treated group, RGE-treated group showed decreased IL-1*β* expression at 7 and 14 days after SCI. (b) Expression of TNF*α* protein in damaged spinal cords before and after SCI. TNF*α* concentration in damaged spinal cord of RGE-treated group was significantly reduced at 7 and 14 days after SCI, compared with that in vehicle-treated control. All values are presented as mean ± SD. *N* = 5. ^*∗∗*^(*p* < 0.01), ^*∗*^(*p* < 0.05) indicate significantly less values than vehicle-treated control.

To identify the effects of RGE on the inflammatory responses in the spinal cord after SCI, we investigated the expression of IL-1*β* and TNF*α* in damaged spinal cord before and after SCI by ELISA (Figure 4). In the normal spinal cord (Pre), IL-1*β* expression was merely detected in both groups, increased in damaged spinal cord after SCI, and peaked at 1 day after SCI in both groups. There was no significant difference in IL-1*β* expression between two groups prior to SCI (Pre) until 3 days after SCI. IL-1*β* expression in the RGE-treated group decreased quickly while that in the vehicle-treated group was kept constant after reaching a peak at 24 hours after SCI. The concentration of IL-1*β* in the RGE-treated group was significantly decreased at 7 and 14 days after SCI when compared to that in the vehicle-treated group (Figure 4(a)). TNF*α* concentration was upregulated in damaged spinal cord after SCI in both groups, and there was no significant difference in TNF*α* level between two groups prior to SCI until 3 days after SCI. At 7 and 14 days after SCI, TNF*α* concentration in damaged spinal cord was significantly decreased in the RGE-treated group compared to the vehicle-treated group (Figure 4(b)). These findings indicated that oral administration of RGE caused significant decreases in the expressions of proinflammatory cytokines, IL-1*β*, and TNF*α*, in the spinal cord at 7 and 14 days after SCI.

**Figure 5:**
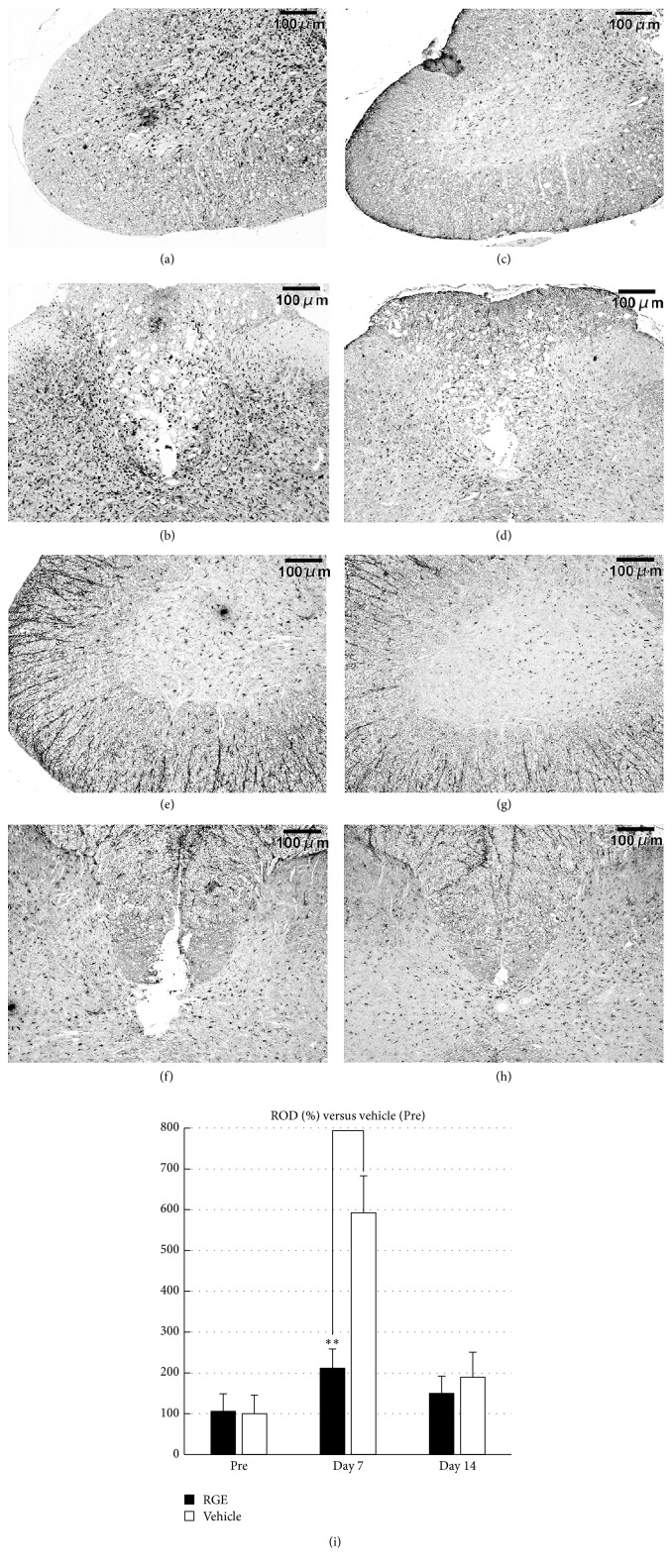
Oral administration of RGE suppressed aggregation of Iba-1-positive microglial cells in injured spinal cord at 7 days after SCI in rats. (a–h) Representative photomicrographs of Iba-1 immunostaining in sections from damaged spinal cord of rats at 7 and 14 days after SCI ((a)–(d): 7 days after SCI; (e)–(h): 14 days after SCI; (a), (b), (e), and (f): vehicle; (c), (d), (g), and (h): RGE (350 mg/kg/day)). Scale bar = 100 *μ*m. (i) Relative optical density (ROD) as % of Iba-1 immunoreactivity in injured spinal cord. There was a significant decrease in Iba-1 immunoreactivity at 7 days after SCI in RGE-treated rats (*n* = 5 in each group). All values are presented as mean ± SD. ^*∗∗*^(*p* < 0.01) indicates significantly lower values than vehicle-treated control.

Both IL-1*β* and TNF*α* are known to activate microglia in the spinal cord and microglia are considered to play a pivotal role in the processes of neuronal degeneration. Therefore, we conducted Iba-1 immunohistochemical staining to demonstrate microglial cells in the spinal cord after SCI. Iba-1-positive cells were observed in both grey and white matter of the spinal cord at 7 and 14 days after SCI. The spinal cord of the vehicle-treated group contained numerous Iba-1-positive cells particularly in the ventral horn (Figure 5(a)), dorsal horn, and dorsal funiculus (Figure 5(b)) at 7 days after SCI. Oral administration of RGE at 350 mg/kg/day caused a marked decline in Iba-1 immunoreactivity within the corresponding areas of the spinal cord at 7 days after SCI (Figures 5(c) and 5(d)). At 14 days after SCI, the distribution pattern of Iba-1-positive cells in the spinal cord of vehicle-treated rats was similar to that in RGE-treated rats (Figures 5(e), 5(f), 5(g), and 5(h)). Quantitative analysis showed a significant decrease in Iba-1 immunoreactivity by RGE treatment at 7 days after SCI (Figure 5(i)).

These experimental results concerning IL-1*β* and TNF*α* expression and Iba-1 immunoreactivity in the injured spinal cord with or without RGE treatment suggest that oral administration of RGE inhibits local inflammatory responses in the rat spinal cord after SCI to attenuate secondary degeneration of the spinal cord tissue involved.

### 3.4. RGE Administration Upregulates Expression of VEGF and Bcl-x_L_ in Spinal Cord after SCI

**Figure 6:**
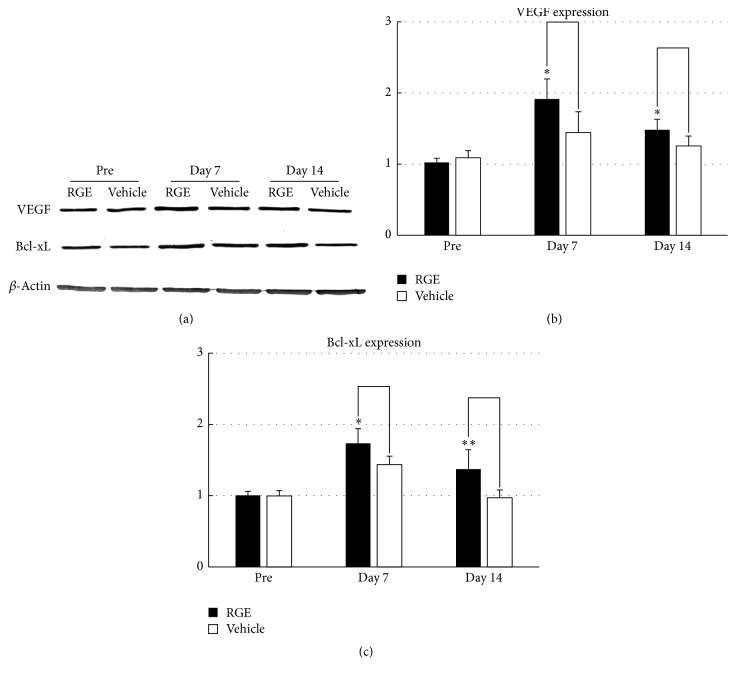
Oral administration of RGE upregulated VEGF and Bcl-x_L_ protein expression in damaged spinal cord at 7 and 14 days after SCI in rats. (a) Representative photographs of Western blot for VEGF and Bcl-x_L_ proteins in spinal cord of rats before SCI and at 7 and 14 days after SCI. ß-Actin was used as an internal control for each sample. ((b) and (c)) Densitometric analyses of VEGF (b) and Bcl-x_L_ (c) immunoreactive bands in spinal cord before SCI and at 7 and 14 days after SCI. Note that VEGF and Bcl-x_L_ protein expressions were upregulated at 7 and 14 days after SCI by treatment with RGE. Data were obtained from five independent experiments. All values are presented as mean ± SD. ^*∗∗*^(*p* < 0.01), ^*∗*^(*p* < 0.05) indicate significantly higher values than vehicle-treated control.

Our previous studies demonstrated that an active ginseng ingredient (ginsenoside Rb_1_) and its chemical derivative, when intravenously infused, can protect neurons from ischemia or compressive injury through upregulation of Bcl-x_L_ and VEGF [9, 14]. Therefore, in this study, we checked whether or not oral administration of RGE could also upregulate the expression of Bcl-x_L_ and VEGF in the spinal cord after SCI. Bcl-x_L_ and VEGF expression was detected by western blotting (Figure 6(a)), and subsequent semiquantitative analyses showed that, at 7 and 14 days after SCI, VEGF and Bcl-x_L_ expression in the spinal cord of the (350 mg/kg/day) RGE-treated group was significantly upregulated when compared to that in the vehicle-treated group (Figures 6(b) and 6(c)). These findings suggest that RGE-induced upregulation of VEGF and Bcl-x_L_ contributes, in part, to prevention of neuronal death in the injured spinal cord.

## 4. Discussion

Oral administration of a ginseng extract at doses of 200 and 500 mg/kg/day was proved to exhibit neuroprotective effects in an MPTP-treated rodent model of Parkinson disease [12]. Based on the above article, we selected 350 mg/kg/day as the high dose of RGE in this study. A one-tenth dose of RGE, namely 35 mg/kg/day, was also selected, because such a low dose of RGE is expected to be effective, at least, for the treatment of ultraviolet-induced cutaneous damage, as demonstrated in our previous study using mice [13]. As a traditional medicine used for thousands of years, RGE was taken by Asia people not only for curing diseases but also for routine health keeping. We started oral administration of RGE from 1 week before SCI to mimic the traditional way of RGE administration. In the present study, the high dose but not low dose of RGE significantly promoted functional recovery from SCI-induced motor and behavioral abnormalities up to levels close to those pre-SCI when orally administered once a day from one week before SCI through 2 weeks after SCI. Since few medicines without any adverse effects are available for prevention, therapy, or treatment of patients with SCI, the present experimental results raise new hope that RGE, even at a rather high dose, if continuously applied orally to patients after SCI and preferably before SCI as well, would alleviate SCI-induced neuronal symptoms. The reasons why the low dose of RGE failed to ameliorate the motor and behavioral abnormalities by 2 weeks after SCI cannot be easily accounted for: possibly degradation of the active ingredient(s) of orally administered RGE in the intestine and/or the blood brain barrier precluded the active ingredient(s) from achieving a maintained local tissue concentration(s) sufficient for recovery of the injured spinal cord. In support of this speculation, one of the putative active ingredients, namely, gRb_1_, has been shown in our previous study to alleviate neural damage by one week after SCI when infused continuously into the blood stream at rather low doses of 12 *μ*g/day (40 *μ*g/kg/day) and 60 *μ*g/day (200 *μ*g/kg/day) [18]. Since continuous intravenous infusion of gRb_1_ appears to be superior to oral administration of RGE in terms of SCI treatment, gRb_1_ rather than RGE would be a candidate drug for the treatment of acute SCI in the future if allowed to be applied to humans. Nevertheless, for the time being, oral administration of RGE, which can be prescribed for humans at least in Asian countries, is likely to be a promising means for SCI treatment. This speculation is reinforced by the present results of MAP2 immunostaining, which demonstrated RGE-mediated suppression of neuronal degeneration in and around the central canal and ventral horn in cases of SCI.

The pathological sequelae after SCI are divided into two consecutive events: primary injury and secondary injury [19, 20]. The direct mechanical trauma to the spinal cord causes primary injury. A series of secondary injury involving ischemia, edema, and inflammation is thought to enlarge the area of cell death through necrosis, apoptosis, and autophagy [20–22]. In the cascade of secondary injury, inflammation is regarded as a key event. Damage to the central nervous system (CNS) including the spinal cord elicits an immediate response of microglia [23] and secretion of proinflammatory cytokines; as a result, inflammation extends in the spinal cord [24]. Among the proinflammatory cytokines, IL-1 is considered to play a pivotal role in neurodegeneration [25]. After SCI, IL-1*α* is rapidly produced by resident microglia at the site of trauma, and upregulation of IL-1*α* in microglia precedes the infiltration into the injured spinal cord of blood-derived innate immune cells, which subsequently amplify local inflammation by producing IL-1*β*, thereby leading to secondary damage to oligodendrocytes. Deletion of the IL-1*α* and IL-1*β* genes reduces recruitment of innate immune cells and protects oligodendrocytes from degenerating after SCI [26]. As a proinflammatory cytokine, TNF*α* has been reported to induce apoptosis in neurons and oligodendrocytes after SCI, therefore initiating the secondary injury in spinal cord [27]. Intraperitoneal injection of TNF*α* antagonist reduced apoptosis of neurons and oligodendrocytes in rat spinal cord injury [28]. The decreased expressions of IL-1*β* and TNF*α* in damaged spinal cord after SCI indicate reduction of the second injury, therefore protecting neuron and oligodendrocyte from apoptosis. Ginsenosides Rd and Re as ingredients of RGE were reported to inhibit inflammation in a novel model of Parkinson's disease [29]; gRb_1_ as a main ingredient of RGE was shown to attenuate microglia-induced neuronal damage [30]; and ginsenoside Rg_3_, a putative metabolite of gRb_1_, was demonstrated to inhibit activation of microglia after systemic LPS treatment [31]. Moreover, oral administration of ginsenoside Rg_1_ as a representative component belonging to protopanaxatriol saponins in RGE was reported to ameliorate TNBS-induced colitis by inhibiting the binding of lipopolysaccharide (LPS) to TLR4 on macrophages and by restoring Th17/Treg balance [32]. These studies concerning the anti-inflammatory actions of RGE ingredients together with the inhibition by RGE of microglial response and the expression of TNF*α* and IL-1*β* in the injured spinal cord, as revealed in the present study, favor the notion that orally administered RGE suppresses SCI-induced tissue degeneration within the white matter and possibly grey matter by attenuating inflammation in situ.

Ischemia in situ has been shown to affect posttraumatic pathological changes in cases of acute SCI as well. To be more specific, it occurs in parallel with neurological dysfunction and aggravates secondary degeneration after SCI. Therefore, to regain blood flow by revascularization is one of the effective ways to protect spinal cord from ischemia after SCI. Secondary injury occurring after SCI was reported to decline in synchrony with revascularization of the involved tissue [33]. In this study, we showed that oral administration of RGE upregulated the expressions of VEGF and Bcl-x_L_ in the injured spinal cord, which were reported to facilitate vascular regeneration and/or neuronal survival in cases of brain ischemia or traumatic neural damage [34, 35]. These results are in accordance with our previous study showing the upregulation of VEGF and Bcl-x_L_ expression by intravenous infusion of gRb_1_ in the injured spinal cord [18].

## 5. Conclusion

The present study showed that oral administration of RGE promotes restoration from motor and behavioral abnormalities after SCI in rats. RGE administration reduced IL-1*β* and TNF*α* expression, suppressed microglial aggregation, and upregulated the expression of VEGF and Bcl-x_L_ in the injured spinal cord, thereby precluding the loss of neurons and facilitating the restoration of white matter in the injured spinal cord at 14 days after SCI. We concluded that orally administered RGE promotes neurorestoration through inhibition of inflammation, and VEGF- and Bcl-x_L_-mediated neovascularization, and cytoprotection in the rat spinal cord insulted with a compressive injury.
